# Altered Neuronal Responses During an Affective Stroop Task in Adolescents With Conduct Disorder

**DOI:** 10.3389/fpsyg.2018.01961

**Published:** 2018-10-18

**Authors:** Lynn V. Fehlbaum, Nora M. Raschle, Willeke M. Menks, Martin Prätzlich, Eva Flemming, Letizia Wyss, Felix Euler, Margaret Sheridan, Philipp Sterzer, Christina Stadler

**Affiliations:** ^1^Department of Child and Adolescent Psychiatry, Psychiatric University Clinics, University of Basel, Basel, Switzerland; ^2^Donders Institute for Brain, Cognition and Behaviour, Radboud University, Nijmegen, Netherlands; ^3^Department of Psychiatry and Psychotherapy, Charité – University Medicine Berlin, Berlin, Germany; ^4^Department of Psychology and Neuroscience, University of North Carolina at Chapel Hill, Chapel Hill, NC, United States

**Keywords:** conduct disorder, emotion processing, response inhibition, amygdala, insula

## Abstract

Conduct disorder (CD) is a psychiatric disorder of childhood and adolescence which has been linked to deficient emotion processing and regulation. The behavioral and neuronal correlates targeting the interaction of emotion processing and response inhibition are still under investigation. Whole-brain event-related fMRI was applied during an affective Stroop task in 39 adolescents with CD and 39 typically developing adolescents (TD). Participants were presented with an emotional stimulus (negative/neutral) followed by a Stroop task with varying cognitive load (congruent/incongruent/blank trials). fMRI analysis included standard preprocessing, region of interest analyses (amygdala, insula, ventromedial prefrontal cortex) and whole-brain analyses based on a 2(*group*) × 2(*emotion*) × 3(*task*) full-factorial ANOVA. Adolescents with CD made significantly more errors, while reaction times did not significantly differ compared to TD. Additionally, we observed a lack of downregulation of left amygdala activity in response to incongruent trials and increased anterior insula activity for CD relative to TD during affective Stroop task processing [cluster-level family-wise error-corrected (*p* < 0.05)]. Even though no three-way interaction (*group* × *emotion* × *task*) interaction was detected, the findings presented still provide evidence for altered neuronal underpinnings of the interaction of emotion processing and response inhibition in CD. Moreover, our results may corroborate previous evidence of emotion dysregulation as a core dysfunction in CD. Future studies shall focus on investigating the interaction of emotion processing and response inhibition in CD subgroups (e.g., variations in callous-unemotional traits, impulsivity, or anxiety).

## Introduction

Conduct disorder (CD) is a psychiatric disorder of childhood and adolescence marked by emotion processing and regulation deficits (American Psychiatric Association, 2013; Fairchild et al., 2013). CD youths typically engage in aggressive and antisocial behavior [e.g., rule breaking, stealing, and lying (Lahey and Waldman, 2012)], present with high rates of comorbidity (Nock et al., 2006), and are at risk for academic failure, delinquency, and mental disorders in adulthood (Swanson et al., 1994; Fergusson et al., 2005; Biederman et al., 2008; Erskine et al., 2016). CD is categorized according to its age of onset (childhood-onset versus adolescent-onset), severity (mild, moderate, or severe), and the presence or absence of callous-unemotional (CU) traits (DSM-5 specifier “with limited prosocial emotions”). Moreover, adolescents with CD can exhibit either predominantly reactive/impulsive or proactive/instrumental aggression, which is associated with the levels of CU traits (Mathias et al., 2007; Freitag et al., 2018). While adolescents with reactive aggression mainly show impulsive behavior, those with proactive aggression usually present with increased CU traits. Together with oppositional defiant disorder (ODD), a milder form and developmental precursor of antisocial behavior, CD has been categorized as a disruptive behavior disorder (DBD) (Loeber et al., 2000). Antisocial youths are phenotypically characterized by a heterogeneous symptomatology, reflected in different etiological paths and variations in response to treatment (Steiner et al., 2017). Main forms of neurocognitive dysfunctions include deficient emotion processing, reduced affective empathy, and altered response inhibition (Blair et al., 2016). Indeed the mechanisms underlying deficient emotion processing and response inhibition have been hypothesized to increase the risk for antisocial behavior (Campbell et al., 2000; Davidson et al., 2000; Young et al., 2009; Wang et al., 2017). As such, a failure to inhibit negative affect and to respond appropriately to negative cues has been proposed to lead to aggressive behaviors (Davidson et al., 2000), as for example observed in CD. Consequently, adolescents with CD have been shown to be impaired when negative cues are presented prior to cognitive task performance, e.g., during response inhibition (Euler et al., 2014; Hwang et al., 2016). In other words, previous findings demonstrate an inability to adequately process distracting emotional information, which results in impaired cognitive performance. Consequently, this may lead to an inability to suppress impulsive and/or antisocial behaviors as observed in CD [similar to observations in family violence (Chan et al., 2010)].

While altered response inhibition has been observed in adolescents with DBD (Prateeksha et al., 2014; Hwang et al., 2016), results are inconsistent in regards to the direction of findings. Some studies measuring response inhibition report no differences in performance of CD or DBD youths (Banich et al., 2007; Rubia et al., 2008, 2010b). Others indicate higher error rates and/or longer reaction times (RTs) (Rubia et al., 2009a; Euler et al., 2014; Prateeksha et al., 2014; Hwang et al., 2016). Importantly, when response inhibition is preceded by emotional (both negative and/or positive) stimuli, decreases in performance are more commonly reported (Euler et al., 2014; Prateeksha et al., 2014; Hwang et al., 2016).

Studies using functional magnetic resonance imaging (fMRI) have shed light on the neuronal phenotype characteristic for CD youths. Most commonly, alterations in neural recruitment in frontal and limbic lobes (including insula, amygdala, and anterior cingulate) are reported (Stadler et al., 2007; Sterzer and Stadler, 2009; Blair, 2010; Rubia, 2011; Raschle et al., 2015; Hwang et al., 2016), which are likely to depend on the levels of CU traits (Blair, 2010; Baker et al., 2015). Previous studies investigating response inhibition (e.g., stop, Simon, switch, or Stroop tasks) in CD have revealed decreased and increased neuronal activity in medial prefrontal cortex, insula, cingulate gyrus, temporoparietal junction, subcortical regions, and occipital lobe (Banich et al., 2007; Rubia et al., 2008, 2009a, 2010b). To our knowledge, only one neuroimaging study has yet directly tested the interaction of emotion processing and response inhibition in DBD youths. In this study, Hwang et al. (2016) detected reduced ventromedial prefrontal cortex (vmPFC) and amygdala activity in response to negative affective stimuli and reduced insula activity with increasing cognitive load in DBD compared to typically developing (TD) youths.

The present study aims at adding to this first evidence in DBD by investigating the neuronal and behavioral correlates of the interaction of emotion processing and response inhibition in CD youths through fMRI during affective Stroop task performance. The affective Stroop task is a variation of a response inhibition task and comprises a number Stroop task with trials which vary in cognitive load. Additionally, negative and neutral images are presented prior to the Stroop trials. By combining emotional images and number Stroop trials, the affective Stroop task allows the investigation of the interaction of emotion processing and response inhibition. In the present study, only adolescents with a clinical diagnosis of CD were included. This is unlike previous studies using a similar task design, but a more lenient DBD diagnosis. We used a task design adapted from Hart et al. (2010), which was previously validated in a sample of healthy young adults (Raschle et al., 2017). In contrast to Hwang et al. (2016), our task included a set of child-appropriate images, reduced task complexity, and adapted presentation times (e.g., shorter image presentation, longer Stroop task presentation). Additional efforts were made in order to develop a protocol that meets the demands of an adolescent sample (e.g., attention and time constraints, see also Raschle et al., 2009, 2012. For further specifications on the task design see section “fMRI Task: The Affective Stroop Task”). Using both region of interest and whole-brain approaches, we hypothesized (I) to observe *emotion* × *task* interactions for the Stroop effect (i.e., delayed RTs for trials with increased cognitive load and prior negative stimulation) in CD compared to TD youths, in line with previous work (Euler et al., 2014); (II) to detect reduced neuronal activity within brain regions involved in emotion processing and response inhibition (amygdala, insula, and vmPFC) during the affective Stroop task in CD relative to TD youths in line with previous findings (Hwang et al., 2016).

## Materials and Methods

### Participants

Seventy-eight youths (39CD/39TD) were included in the present analyses (age range: 10.1–19.1 years, mean age: 15.7 years, 10 males in each CD/TD group were assessed in Berlin). CD was diagnosed according to DSM-5 criteria by trained PhD students. Seventeen CD youths (43.6%) additionally met DSM-5 criteria for present attention-deficit hyperactivity disorder, while 20 CD youths (51.3%) additionally met diagnostic criteria for ODD. TD were included if no current psychiatric diagnosis was reported by either the participant and/or the parents/legal guardians. CD and TD groups were matched for age [*t*(76) = 0.87, *p* = 0.390] and non-verbal IQ [*t*(76) = -0.72, *p* = 0.472] (**Table 1** and see section **Supplementary Material** – Additional Information on Participants”). Moreover, both CD and TD groups from Basel and Berlin did not differ in age [CD: *t*(37) = 1.04, *p* = 0.303; TD: *t*(37) = -0.92, *p* = 0.365] or non-verbal IQ [CD: *t*(37) = 1.12, *p* = 0.269; TD: *t*(37) = 1.47, *p* = 0.151]. Participants were recruited through referrals from child and adolescent psychiatric institutions, public schools, and the general public through the use of fliers.

**Table 1:** Behavioral group characteristics.

		CD	TD	*p*
		Mean ± SD	Mean ± SD	Significance two-tailed
		*N* = 39	*N* = 39	
Age (in years)		15.94 ± 1.88	15.54 ± 2.15	0.390
Sex (male/female)		29/10	29/10	
No. per site (Basel/Berlin)		28/11	28/11	
Handedness (right/left/ambidextrous)		36/2/1	36/2/1	
IQ	Matrix reasoning	99.47 ± 12.02	101.54 ± 11.31	0.472
Comorbidities (DSM-5)	Attention-deficit hyperactivity disorder	17	0	
	Oppositional defiant disorder	20	0	
	Major depression	2	0	
	Anxiety disorder	6	0	
	Substance/alcohol abuse/dependence	16	0	
YPI		*N* = 39	*N* = 38	
	Grandiose-manipulative (interpersonal) dimension	8.68 ± 2.74	7.89 ± 1.90	0.117
	Callous-unemotional (affective) dimension	11.23 ± 3.08	9.55 ± 1.94	0.006^∗∗^
	Impulsive-irresponsible (behavioral) dimension	13.57 ± 2.80	10.68 ± 1.72	<0.001^∗∗∗^
	Total score	11.16 ± 2.38	9.37 ± 1.21	<0.001^∗∗∗^

*^∗∗^*p* < 0.01; ^∗∗∗^*p* < 0.001, two-tailed *t*-test; all other *t*-tests non-significant at threshold *p* = 0.05. For IQ, standard scores are reported; for YPI, mean scores are reported, missing data are due to time constraints. CD, conduct disorder patients; TD, typically developing adolescents; SD, standard deviation; YPI, Youth Psychopathic traits Inventory.*

### Ethics Statement

All adolescents and parents/legal guardians gave written informed consent as approved by the local ethics committee ‘Ethikkommission der Nordwest- und Zentralschweiz’ and received vouchers for their participation.

### Clinical Testing and Questionnaires

Conduct disorder youths and their legal guardians completed the Schedule for Affective Disorders and Schizophrenia for School-Age Children – Present and Lifetime version (K-SADS-PL; Kaufman et al., 1997) in order to assess CD criteria and comorbid disorders according to the DSM-5 (**Table 1**). CD and TD participants completed the Youth Psychopathic traits Inventory (YPI, Andershed et al., 2007) and the matrix reasoning subtest of the WISC-IV (ages ≤ 16 years; Petermann, 2011) and the WAIS-III (ages ≥ 17 years; Petermann, 1997) measuring non-verbal IQ. For 10 participants (9 CD and 1 TD), only a composite IQ score was obtained. CD and TD legal guardians moreover completed a socioeconomic status (SES) questionnaire (see section “**Supplementary Material** – Psychometric Testing: Socioeconomic Status”). Participants were asked to report any medication administered prior to the MRI session (see section “**Supplementary Material** – Medication of Adolescents With Conduct Disorder (*N* = 35) and Typically Developing Controls (*N* = 39) at MRI Session”).

### fMRI Task: The Affective Stroop Task

**FIGURE 1:**
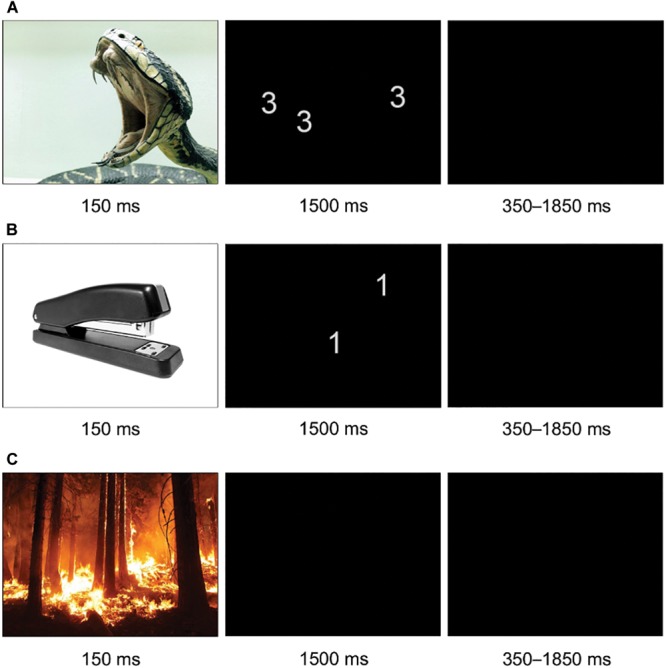
fMRI task design including three exemplary affective Stroop trials. **(A)** Negative-congruent trial; **(B)** neutral-incongruent trial; **(C)** negative-blank trial.

We applied an affective number Stroop task as previously described in Raschle et al. (2017) (**Figure 1**). Each trial started with an emotional stimulus, i.e., a negative (Neg) or neutral (Neu) stimulus (150 ms), followed by a task trial (congruent/incongruent/neutral Stroop trial or a blank screen) and finally a relaxation period, i.e., blank screen (350 ms). All pictures were selected from a child-appropriate image system [Developmental Affective Photo System (DAPS); Cordon et al., 2013]. During task trials, participants were presented with an array of 1 to 4 digits or a blank screen and were asked to press a button corresponding to the number of items displayed. The number of items was either congruent (C; e.g., number 3 in an array of 3) or incongruent (IC; e.g., number 1 in an array of 2) with the digits presented. Star shaped stimuli (S; as a neutral baseline counting condition) and blank trials (B; no response expected from participants) were used as control conditions (for further details see Raschle et al., 2017). Trial order and interstimulus intervals (which were 350–1850 ms) were randomized using Optseq^1^ and kept constant across participants. A total of 300 task and 100 blank trials were administered (100 for C/IC/S trials, 50 with preceding negative images, 50 with neutral images, in 2 runs), with a total scan time of about 16 min (7.59 min each run).

### Behavioral Measures: In-Scanner Performance

All participants scored > 60% correct responses per task condition and run. RTs and task accuracy (raw scores) were analyzed using 2x2x2 full-factorial ANOVAs with the between-subject factor *group* (CD and TD) and within-subject factors *emotion* (negative and neutral) and *task* (congruent and incongruent) for RTs and accuracy separately using SPSS, version 24. Data was unavailable for a minority of responses because of technical difficulties with the response box (for a detailed description see section “**Supplementary Material** – Button Box Responses”).

### fMRI Data Acquisition and Analysis

#### Acquisition Parameter

In Basel, whole-brain blood oxygen level-dependent (BOLD) fMRI data and structural T1-weighted magnetization prepared rapid gradient echo imaging images were acquired on a Siemens 3T Prisma MRI system using a 20-channel phased-array radio frequency head coil. In Berlin, a Siemens 3T TimTRIO MRI system equipped with a 12-channel head coil was used. At both sites a T2^∗^-weighted EPI (echo-planar imaging) sequence with TR = 2000 ms, TE = 30.0 ms, FOV = 192 mm, image matrix = 64 mm × 64 mm, voxel size = 3 mm × 3 mm × 3 mm, and number of slices = 37 was used. We further acquired high-resolution T1-weighted structural images for coregistration during fMRI preprocessing using the following specifications: TR = 1900.0 ms, TE = 3.42 ms, FOV = 256 mm, image matrix = 256 × 256, voxel size = 1 mm.

#### fMRI Analysis

Functional MRI data were analyzed using the Statistical Parametric Mapping software, version 12 (SPM12 ^2^). Preprocessing of the data included realignment, co-registration to the structural image, segmentation, normalization to the Montreal Neurologic Institute (MNI) standard brain, and spatial smoothing using an 8 mm Full Width at Half Maximum Gaussian kernel.

Single-subject fMRI data was analyzed using the general linear model. The model comprised eight task regressors [each combining a negative or neutral stimulus with congruent, incongruent, or neutral (stars/blank) Stroop trials, namely negative-congruent (NegC), negative-stars (NegS), negative-incongruent (NegIC), negative-blank (NegB), neutral-congruent (NeuC), neutral-stars (NeuS), neutral-incongruent (NeuIC), neutral-blank (NeuB)], one regressor for incorrect/missed responses, and six motion regressors. The task regressors were modeled as stick functions convolved with the hemodynamic response function as implemented in SPM12. Stars trials were not considered in further analyses.

At the second level, hypothesis-based ROI and whole-brain analyses were performed. *A priori* defined anatomical ROIs included bilateral amygdala, insula, and vmPFC based on previous literature (Rubia et al., 2008, 2009a, 2010a; Rubia, 2011; Raschle et al., 2015; Hwang et al., 2016) and derived from the automated anatomical labeling atlas (aal; Tzourio-Mazoyer et al., 2002). Mean parameter estimates were extracted from each ROI using the marsbar toolbox (Brett et al., 2002). A repeated measures ANOVA with the factors *group* (CD and TD), *emotion* (negative and neutral), and *task* (blank, congruent, and incongruent) and follow-up pairwise comparisons applying a Bonferroni multiple comparisons correction in order to account for the number of ROIs were then computed within SPSS, version 24.

For whole-brain analyses, beta images resulting from first-level model estimation for each regressor and run were submitted to a group-level random-effects analysis using a 2x2x3 full-factorial ANOVA with the between-subject factor *group* (CD and TD) and within-subject factors *emotion* (negative and neutral) and *task* (blank, congruent, and incongruent).

Quality control was performed throughout the analyses in order to control for effects of motion. Besides including six additional motion regressors during single-subject analysis, each analysis mask was visually inspected for head motion. An *F*-test performed on the six motion regressors revealed no significant differences in quantitative motion between groups [*F*(5,72) = 0.34, *p* = 0.889]. Qualitative motion was evaluated using Artifact Detection Tools (Whitfield-Gabrieli, 2009), where excessive motion was defined by the number of scans with a movement threshold of >2 mm and a rotation threshold of >0.05 mm. A two-sample *t*-test was then conducted, resulting in no significant differences in qualitative motion across groups [*t*(74) = 0.49, *p* = 0.626]. For all analyses, *site* (Basel, Berlin) was added as an additional factor of no interest. Brain activation was assessed for the main effects of *group, emotion*, and *task*, and all possible interactions thereof are reported at a cluster-extent family-wise error (FWE) rate of *p* < 0.05 (cluster building threshold of *p* < 0.001). Significant clusters of main effects and interactions were followed up with masked *post hoc t*-tests.

The *F*-maps and *t*-maps of the whole-brain analyses are available at https://neurovault.org/collections/XEYAPOGU/ (Gorgolewski et al., 2015) and the mean parameter estimates from each ROI are available in the **Supplementary Material**.

## Results

### Questionnaires

Psychometric assessments are reported in **Table 1**. CD scored significantly higher than TD in the callous-unemotional (i.e., affective) and impulsive-irresponsible (i.e., behavioral) dimensions and the total score of the YPI (all *p* < 0.01; YPI, Andershed et al., 2007). Nevertheless, psychopathic traits in our CD group were overall low [YPI total score: *M* = 11.16 (maximum score: 20.00), *SD* = 2.38, CU dimension: *M* = 11.23 (maximum score: 20.00), *SD* = 3.08; both within one standard deviation of a representative school sample (*N* = 480), see also Stadlin et al., 2016].

### Behavioral Results: In-Scanner Performance

Analysis of RTs revealed a significant main effect of *emotion* [Neu > Neg, *F*(1,76) = 5.74, *p* < 0.05], and a main effect of *task* [IC > C, *F*(1,76) = 615.48, *p* < 0.001]. There was no main effect of *group* and no interaction effects for RTs. For accuracy, we found a significant main effect of *emotion* [Neu > Neg, *F*(1,76) = 8.29, *p* < 0.01], a main effect of *task* [C > IC, *F*(1,76) = 118.42, *p* < 0.001], and a main effect of *group* [CD < TD, *F*(1,76) = 6.77, *p* < 0.05]. There were no significant interaction effects for accuracy [see section “**Supplementary Material** – In-Scanner Performance (Accuracy and Reaction Times) for Adolescents With Conduct Disorder (*N* = 39) and Typically Developing Controls (*N* = 39)].

### Functional MRI Results

#### ROI Results

**FIGURE 2:**
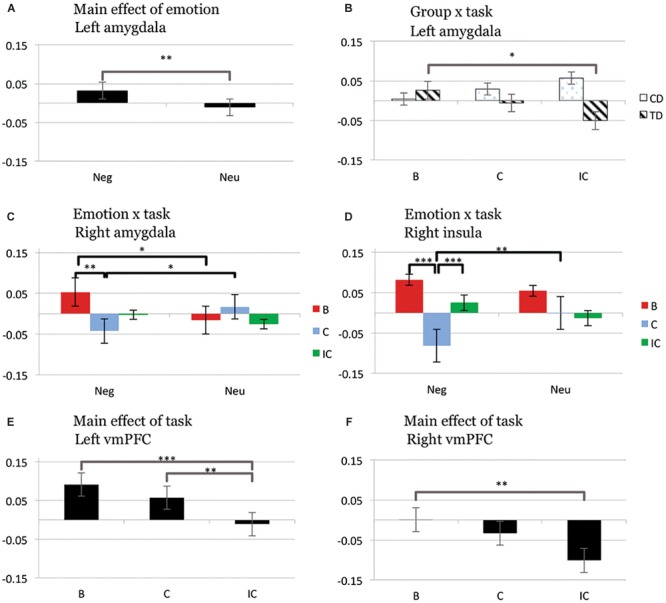
Bar graphs displaying mean values of parameter estimates (mean centered) in predefined regions of interest [amygdala, insula, ventromedial prefrontal cortex (vmPFC)] for the main effect of *emotion* and *group* × *task* interaction (left amygdala; **A,B**), *emotion* × *task* interaction (right amygdala and insula; **C,D**), and main effect of *task* (left and right vmPFC; **E,F**). B, blank trial; C, congruent trial; IC, incongruent trial; Neg, negative trial; Neu, neutral trial; CD, conduct disorder; TD, typically developing adolescents, ^∗^*p* < 0.05; ^∗∗^*p* < 0.01; ^∗∗∗^*p* < 0.001, two-tailed *t*-test; all other *t*-tests non-significant at threshold *p* = 0.05.

ROI analyses in relevant regions of interest (bilateral amygdala, insula, and vmPFC) revealed significant *group* × *task* and *emotion* × *task* interactions, as well as main effects of *emotion* and *task* (**Figure 2**).

##### Group × task interaction

A *group* × *task* interaction was observed in left amygdala [*F*(2,73) = 4.83, *p* < 0.05], reflecting significantly decreased activity for incongruent compared to blank trials in TD (IC < B, *p* < 0.05), but not CD (all *p* > 0.227). This effect was independent of emotion [no significant *group* × *emotion* × *task* interaction; *F*(2,73) = 0.73, *p* = 0.485]. After exclusion of participants with medication or substance/alcohol abuse or dependence the *group* × *task* interaction in left amygdala (main finding) remained significant [*F*(2,57) = 3.54, *p* < 0.05]. Similarly, inclusion of the YPI CU dimension as a covariate did not change the significance levels of the *group* × *task* interaction in left amygdala [*F*(2,71) = 4.52, *p* < 0.05].

In order to examine the relationship between psychopathic traits (YPI total score) and left amygdala activity during IC–C (*group* × *task* interaction), follow-up bivariate correlations were computed for CD and TD separately. Results revealed no significant relationships between left amygdala activation and psychopathic traits for CD or TD.

##### Emotion × task interaction

A significant *emotion* × *task* interaction effect was found in right amygdala [*F*(2,73) = 4.77, *p* < 0.05]. Across all subjects we observed relatively increased right amygdala activity for blank trials with a prior negative compared to neutral emotion (NegB > NeuB, *p* < 0.05), but relatively decreased activity in the right amygdala during congruent trials with a prior negative versus neutral emotion (NegC < NeuC, *p* < 0.05). In addition we observed decreased activity in the right amygdala for congruent relative to blank trials following negative stimuli (NegC < NegB, *p* < 0.01). Within right insula, a significant *emotion* × *task* effect was observed [*F*(2,73) = 5.40, *p* < 0.01]. This effect reflected increased activity during congruent trials following negative compared to neutral stimuli (NegC > NeuC, *p* < 0.005) and increased activity for incongruent compared to congruent trials after negative stimuli (NegIC > NegC, *p* = 0.001). Decreased right insula activity was moreover detected for congruent compared to blank trials following negative stimuli (NegC < NegB, *p* < 0.001).

##### Main effect of emotion

A significant main effect of *emotion* [*F*(1,74) = 7.12, *p* < 0.01] was detected in left amygdala, driven by increased neuronal activity for negative compared to neutral trials (Neg > Neu, *p* < 0.01).

##### Main effect of task

A significant main effect of *task* was detected in right [*F*(2,73) = 5.40, *p* < 0.01] and left vmPFC [*F*(2,73) = 9.36, *p* < 0.001], resulting from relatively decreased activation for incongruent compared to both blank and congruent trials (IC < B, *p* < 0.001; IC < C, *p* < 0.005) for the left hemisphere and incongruent compared to blank trials for the right hemisphere (IC < B, *p* < 0.005).

#### Whole-Brain Results

Whole-brain analysis of brain activation during affective Stroop task processing revealed significant main effects of *group* and *task* (**Table 2**), but no significant main effect of *emotion.* There were no significant two-way or three-way interaction effects. Whole-brain findings did not overlap with the ROI results. All images are neurologically displayed using the Multi-image Analysis GUI as available at http://ric.uthscsa.edu/mango/mango.html.

**Table 2:** Montreal Neurologic Institute (MNI) coordinates, cluster size and Z-scores for whole-brain results using a FWE cluster level correction of *p* < 0.05 (cluster building threshold of *p* < 0.001) for the main effect of *group* and main effect of *task* during the affective Stroop task.

Brain region	Hem	k	Z_0_	MNI coordinates [mm]		
				x	y	z
**Main effect of *group***						
**CD < TD**						
Supramarginal gyrus, middle frontal gyrus, including insula and precentral gyrus	R	1035	7.19	40	–10	20
Postcentral gyrus	L	392	6.04	–62	–2	26
Middle/superior temporal gyrus, hippocampus	R	335	6.66	50	–18	–4
Pallidum, thalamus	R/L	309	6.68	16	–16	–2
**CD > TD**						
Inferior/superior parietal lobe, middle temporal/occipital lobe	R/L	4310	6.27	–30	–82	26
Precentral gyrus, inferior orbitofrontal lobe, caudate, putamen, including insula	R/L	3869	>8	–30	12	22
Rolandic operculum, inferior parietal lobe	L	2545	7.71	–32	–44	–26
Inferior/middle/superior frontal lobe, precentral gyrus, insula	R	1680	7.44	48	34	28
Lingual gyrus, hippocampus, inferior temporal/occipital lobe, cerebellum	R/L	1114	7.04	32	–54	8
Middle/superior frontal gyrus, supplementary motor area, anterior/middle cingulate gyrus	R/L	787	6.28	4	26	44
Anterior/middle cingulate gyrus, caudate	R/L	391	6.45	4	–2	32
Precentral gyrus, superior frontal gyrus	L	343	6.29	–28	–24	30
Inferior parietal lobe, angular gyrus	R	339	5.73	54	–34	18
Fusiform gyrus, inferior/middle occipital lobe	L	294	6.00	–46	–82	–8
Fusiform gyrus, inferior/middle occipital lobe	R	257	5.81	42	–52	–14
Supplementary motor area, superior frontal lobe	L	212	5.78	–18	14	62
**Main effect of *task***						
**IC > C**						
Calcarine sulcus, lingual gyrus, superior occipital lobe	R/L	385	4.23	4	–82	0
**IC < C**						
No suprathreshold voxels						
**IC > B**						
Occipital lobe, fusiform gyrus, calcarine sulcus, cerebellum	R/L	9665	>8	34	–86	0
Supramarginal gyrus, inferior/superior parietal lobe, middle/superior frontal lobe	L	4888	>8	–46	–36	58
Supramarginal gyrus, inferior/superior parietal lobe	R	1118	5.57	42	–40	52
Hippocampus, pallidum, putamen, amygdala	L	791	5.53	–24	0	–8
Inferior frontal operculum, precentral gyrus	L	291	5.82	–54	8	38
Pallidum, caudate, putamen	R	261	4.74	26	6	–8
**IC < B**						
Middle/posterior cingulate gyrus, paracentral lobule, including precuneus	R/L	1850	5.98	–2	–34	44
Inferior/middle temporal lobe, inferior parietal lobe, middle occipital lobe	L	872	6.67	–40	–84	28
Middle frontal lobe, precentral gyrus	R	832	4.95	36	–16	44
Middle/superior temporal lobe, angular gyrus	R	645	4.65	42	–80	30
Inferior temporal lobe, including fusiform gyrus	L	232	4.70	–28	–36	–18
**C > B**						
Supramarginal gyrus, inferior/superior parietal lobe, superior frontal lobe	L	3740	>8	–44	–38	60
Cerebellum, occipital lobe, including fusiform gyrus	R	3049	6.71	32	–88	0
Cerebellum, occipital lobe, including fusiform gyrus	L	2248	6.99	–38	–90	–8
Supramarginal gyrus, inferior/superior parietal lobe	R	747	4.95	44	–40	58
Hippocampus, putamen	L	232	4.11	–18	0	14
**C < B**						
Middle cingulate gyrus, precuneus, paracentral lobule	R/L	941	4.96	6	–38	50
Postcentral gyrus, precentral gyrus	R	432	4.17	36	–20	56
Angular gyrus, middle/superior occipital lobe	L	455	4.81	–44	–82	22
Middle/superior temporal lobe	R	312	4.09	64	–52	6
Fusiform gyrus, lingual gyrus	R	228	4.48	28	–44	–12
Insula, putamen, rolandic operculum	R	221	4.06	36	–12	6

*Family-wise error cluster level correction of *p* < 0.05 (cluster building threshold of *p* < 0.001). Hem, hemisphere; k, cluster size; B, blank trial; C, congruent trial; IC, incongruent trial; CD, conduct disorder; TD, typically developing adolescents.*

##### Main effect of group

**FIGURE 3:**
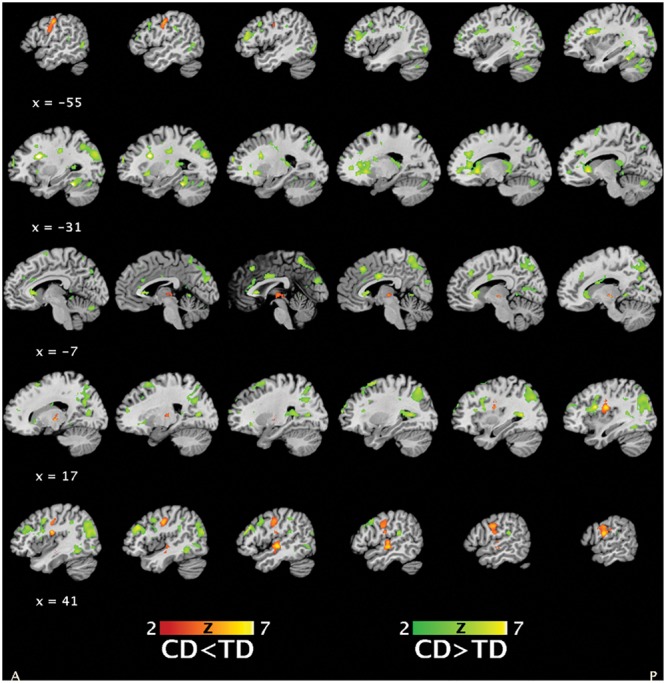
Statistical parametric maps depicting the main effect of *group* (masked *post hoc t*-tests for group differences in brain activation between conduct disorder (CD) and typically developing (TD) adolescents, 39CD/39TD; hypoactivations in CD in red-yellow, hyperactivations in CD in green-yellow) (*p* < 0.05, FWE).

A main effect of *group* (**Figure 3**) was detected for regions including bilateral parietal and middle/inferior temporal and occipital lobes, bilateral precentral and inferior orbitofrontal areas extending into anterior insula and striatum, frontal cortices, and anterior/middle cingulate cortex (CD > TD) and regions including right middle frontal and supramarginal gyri (extending into posterior insula), left postcentral gyrus, right middle/superior temporal cortex, and bilateral thalamus (CD < TD).

##### Main effect of task

Regions showing a differential BOLD response in response to *task* included bilateral parietal and frontal lobes, supramarginal gyri, occipital, temporal, and cerebellar regions, right middle cingulate cortex, left precuneus, and left amygdala. Bilateral supramarginal, superior frontoparietal, and occipital areas exhibited increased activity for congruent and incongruent relative to blank trials (IC/C > B). Left amygdala and inferior frontal areas exhibited increased activity for incongruent compared to blank trials (IC > B). In contrast, decreased left inferior parietal lobe, right middle frontal and cingulate cortices, and left precuneus activity was detected for congruent and incongruent relative to blank trials (C/IC < B). Decreased activity in left inferior temporal and right middle/superior temporal regions was related to incongruent versus blank trials (IC < B, **Supplementary Figure 1**).

## Discussion

Here, we aimed at investigating the interaction of emotion processing and response inhibition in CD youths during an affective Stroop task. Behaviorally, adolescents with CD made significantly more errors, while RTs did not significantly differ compared to TD. ROI analyses revealed a significant *group* × *task* interaction effect reflecting a lack of downregulation of left amygdala activity in response to incongruent task trials for CD compared to TD. This effect was independent of the emotion presented prior to Stroop task performance. Additionally, whole-brain analyses revealed a significant main effect of *group* representing increased anterior insula activity for CD relative to TD regardless of emotion and task demands.

Contrary to our hypothesis and some previous investigations (Rubia et al., 2009b; Euler et al., 2014; Prateeksha et al., 2014) we did not detect group differences in RTs. However, research has not been conclusive to date and the present finding is in accordance with other studies (Banich et al., 2007; Rubia et al., 2008, 2009a, 2010b). Increased RTs for neutral compared to negative trials and for incongruent compared to congruent trials were detected across all participants. While increased RTs robustly reflect the Stroop effect (Stroop, 1935), shorter RTs for negative compared to neutral stimuli were not expected. However, participants’ responses were more accurate after presentation of neutral relative to negative images. Faster responses at the expense of lower accuracy may be due to heightened stress-related anxiety (Panayiotou and Vrana, 2004; Kosinski, 2008). Moreover, in line with Rubia et al. (2009a), CD youths made more errors than TD, which is contrary to other reports in DBD and TD (Banich et al., 2007; Rubia et al., 2008, 2010b; Euler et al., 2014; Prateeksha et al., 2014).

In line with our second hypothesis, ROI analyses revealed decreased left amygdala activity during Stroop task trials with a high cognitive load (IC < B) in TD. In contrast, CD youths did not show any downregulation of emotion-related brain areas with increasing task difficulty. Unexpectedly, this group difference was independent of the emotionality. We would have expected to detect a difference depending on the emotion presented (i.e., a downregulation after negative images instead of on any image as observed here). Our data suggests that no task-dependent downregulation of left amygdala response takes place in CD as compared to TD, possibly reflecting altered neuronal functioning of left amygdala which may be linked to altered regulatory processes.

In agreement with the findings presented here, an aberrant amygdala activity in DBD has previously been reported during response inhibition (Hwang et al., 2016), facial emotion processing (Marsh et al., 2008; Jones et al., 2009; Holz et al., 2017), emotion processing (Sterzer et al., 2005), stimulus-reinforcement learning, and reward processing (Finger et al., 2011). However, past results are inconsistent regarding the direction of findings. Some studies have detected decreases in amygdala activity during emotion processing in adolescents with CD. These findings have partly been attributed to the presence of CU or psychopathic traits (Marsh et al., 2008; Jones et al., 2009; Finger et al., 2011; Hwang et al., 2016), while others have detected amygdala activity reductions in youths with CD without taking callousness into account (Sterzer et al., 2005; Holz et al., 2017). In contrast, another body of work has reported increased amygdala activity during emotion processing tasks in individuals with CD, especially in those with low CU traits or increased anxiety (Herpertz et al., 2008; Decety et al., 2009; Viding et al., 2012; Sebastian et al., 2014). Additionally, it has been postulated that increased amygdala activity might be related to reactive aggression, which is commonly reported in adolescents with CD and low CU traits (Blair, 2010). These individuals are further often characterized by high anxiety levels linked to a hypersensitive threat system and increased sensitivity to negative stimuli. Altogether, increased anxiety and sensitivity to aversiveness of the adolescents with CD studied here may represent a potential reason for a lacking downregulation of amygdala response as observed here.

In addition to functional MRI evidence, past research has suggested reduced amygdala volumes in adolescents with conduct problems (Sterzer et al., 2007; Huebner et al., 2008; Fairchild et al., 2011; Wallace et al., 2014; Rogers and De Brito, 2016). Together with the findings presented here, evidence thus supports a broader view of the amygdala as a key center of alterations in CD.

Whole-brain results provided further insights into insula activity during the affective Stroop task while distinguishing between insular subdivisions. CD exhibited increased activity in anterior insula implicated in affective and cognitive processing (Mutschler et al., 2009; Chang et al., 2013). Additionally, CD showed decreased activity in posterior insula, an area involved in sensorimotor processing (Mutschler et al., 2009; Chang et al., 2013). Our observations are in line with a broader view of the insula in integrating emotion and cognition in healthy adolescents (Chang et al., 2013; Pavuluri and May, 2015), whereas alterations thereof could be hypothesized to reflect an increased allocation of neuronal resources for emotion and cognitive processing, potentially related to a maturational delay in youths with CD. However, the observed differences emerged from a main effect of *group* and therefore need to be interpreted carefully. Future studies shall determine whether right amygdala and insula show significant co-activations (Kober et al., 2008) during task trials following negative stimuli, reflecting on the role of the insula in transferring sensory information to the amygdala (Shelley and Trimble, 2004). In this regard, previous evidence has demonstrated decreased functional connectivity between amygdala and insula in adolescents with disruptive behavior both with (Finger et al., 2012) and without (Hwang et al., 2016) increased CU traits. Overall, alterations in connectivity between amygdala and insula have been observed in a variety of psychiatric disorders linked to emotion processing and regulation deficits such as attention-deficit hyperactivity disorder (Hulvershorn et al., 2014), high-functioning autism spectrum disorder (Ebisch et al., 2011), depression (Bebko et al., 2015), post-traumatic stress syndrome (Rabinak et al., 2011), or generalized anxiety disorder (Roy et al., 2013).

### Limitations

For the present study design we used child-appropriate negative and neutral pictures (DAPS; Cordon et al., 2013). However, the short presentation (150 ms) and moderate image valence might have resulted in a reduced impact for CD youths. Also, the use of static images might not represent an ideal ecologically valid method to investigate adolescents’ response to emotional situations, despite of their common use in affective neuroscience studies. Future studies might consider the use of emotional video clips [e.g., “The Champ,” commonly used for emotion induction (Gross and Levenson, 1995; Hewig et al., 2005)] to overcome this limitation. Moreover, we cannot exclude that confounding factors or comorbidities could have influenced the results. Additionally, the results presented here characterize a group of CD youths on the lower spectrum of CU traits. Interpretation should therefore be drawn with caution. Finally, behavioral and neuronal analyses revealed no three-way interactions (*group* × *emotion* × *task*). This is likely due to the intricate nature of the interaction of emotion processing and response inhibition, which has proven to be very challenging to capture. This is also reflected in earlier studies. For example, a previous study with similar aims neuronally reported a *group* × *emotion* × *task* interaction within the superior frontal gyrus and caudate, however, no such effect was detected in the behavioral analyses (Hwang et al., 2016). Nevertheless, our results are in favor of previous findings of an altered interaction of emotion processing and response inhibition in CD.

## Conclusion

We here provide evidence for the neuronal characteristics of the interaction of emotion processing and response inhibition interaction in CD. More specifically, we observed a significant lack of downregulation of left amygdala activity in response to incongruent task trials and increased anterior insula activity for CD relative to TD during affective Stroop task performance. Behaviorally, CD scored significantly lower than TD youths, while RTs did not differ. While no three-way interactions (*group* × *emotion* × *task*) were detected, our results still support previous findings of an altered interaction of emotion processing and response inhibition in CD. Overall, the present findings extend knowledge on the neurocognitive mechanisms in CD youths and support emotion dysregulation as a core deficit in CD. Future studies shall focus on investigating the interaction of emotion processing and response inhibition in CD subgroups (e.g., variations in CU traits, impulsivity, or anxiety). A deeper characterization of adolescents with CD is particularly relevant as a diagnosis of CD in adolescence represents a risk factor for psychiatric disorders such as antisocial personality disorder or substance use disorders in later adolescence and adulthood (Fergusson et al., 2005; Biederman et al., 2008). Early prevention and targeted interventions may reduce the individual and societal burden associated with a diagnosis of CD.

## Author Contributions

NR, PS, and CS: conception and design of the experiments. LF, WM, MP, EF, LW, and FE: data collection. LF, NR, PS, and CS: data analysis and interpretation. LF, NR, PS, and CS: drafting the paper. LF, NR, WM, MP, EF, LW, FE, MS, PS, and CS: revision and final approval of the version to be published.

## Conflict of Interest Statement

The authors declare that the research was conducted in the absence of any commercial or financial relationships that could be construed as a potential conflict of interest.

## Footnotes

1 http://surfer.nmr.mgh.harvard.edu/optseq

2 www.fil.ion.ucl.ac.uk/spm/
