# Supplementary material for: Altered Neuronal Responses During an Affective Stroop Task in Adolescents With Conduct Disorder
Source: Front Psychol. 2018 Oct 18;9:1961. doi: 10.3389/fpsyg.2018.01961 (PMC6200838; doi:10.3389/fpsyg.2018.01961)
Supplement: Supplementary file 2 [file Data_Sheet_1.pdf]

Supplementary Material

Altered Neuronal Responses During an Affective Stroop Task in Adolescents With Conduct Disorder

Lynn Valérie Fehlbaum*, Nora Maria Raschle, Willeke Martine Menks, Martin Prätzlich, Eva Flemming, Letizia Wyss, Felix Euler, Margaret Sheridan, Philipp Sterzer, & Christina Stadler

*** Correspondence:** Lynn V. Fehlbaum: Lynn.Fehlbaum@upk.ch

1 Additional Information on Participants

All adolescents were invited to take part in two separate sessions including psychometric testing and magnetic resonance imaging. Clinical and behavioral assessments were conducted at the Department of Child and Adolescent Psychiatric Center in Basel or at the Department of Psychiatry and Psychotherapy at Charité – Universitätsmedizin Berlin, Campus Mitte, while neuroimaging took place at the University Hospital of Basel or at the Berlin Center of Advanced Neuroimaging at the Charité – Universitätsmedizin Berlin. We excluded subjects with an IQ score below 70. Adolescents with conduct disorder (CD) were assessed using the Schedule for Affective Disorders and Schizophrenia for School-Age Children–Present and Lifetime version (K-SADS-PL, Kaufman et al. (1997) and were excluded if they did not meet the DSM-5 diagnostic criteria for CD.

Kaufman, J., Birmaher, B., Brent, D., Rao, U., Flynn, C., Moreci, P., . . . Ryan, N. (1997). Schedule for Affective Disorders and Schizophrenia for School-Age Children-Present and Lifetime Version (K-SADS-PL): initial reliability and validity data. *Journal of the American Academy of Child and Adolescent Psychiatry, 36*(7), 980-988. doi:10.1097/00004583-199707000-00021

2 Psychometric Testing: Socioeconomic Status

Conduct disorder (CD) and typically developing (TD) adolescents did not differ in socioeconomic status as indexed by mothers' education and family income earned within the last 12 months. However, they did differ with regards to fathers' education (U=298.00, p=0.017). Furthermore, CD and TD did not differ in subjective social status as assessed by the MacArthur Community ladder measuring social status within their community, but did differ in the MacArthur SES ladder assessing social status within their country (U=196.50, p=0.026).

		CD	TD	*p*	
		[%]	[%]	**Sig. 2-tailed**	
				Mann-Whitney U test	
**Mother characteristics**					
	N=	28	38		
**Education (highest degree earned)**	Pre-primary education (ISCED 0)	0.00%	0.00%	0.356	
	Primary education or first stage of basic education (ISCED 1)	3.60%	2.60%		
	Lower secondary or second stage of basic education (ISCED 2)	14.30%	5.30%		
	(Upper) secondary education (ISCED 3)	60.70%	65.80%		
	Post-secondary non-tertiary education (ISCED 4)	3.60%	5.30%		
	First stage of teritary education (ISCED 5)	17.90%	21.10%		
	Second stage of tertiary education (doctoral level) (ISCED 6)	0.00%	0.00%		
**Father characteristics**					
	N=	24	37		
**Education (highest degree earned)**	Pre-primary education (ISCED 0)	4.20%	0.00%	0.017	*
	Primary education or first stage of basic education (ISCED 1)	4.20%	0.00%		
	Lower secondary or second stage of basic education (ISCED 2)	4.20%	5.4%		
	(Upper) secondary education (ISCED 3)	62.50%	37.80%		
	Post-secondary non-tertiary education (ISCED 4)	0.00%	2.70%		
	First stage of tertiary education (ISCED 5)	25.00%	54.10%		
	Second stage of tertiary education (doctoral level) (ISCED 6)	0.00%	0.00%		
**Family characteristics**					
	N=	23	33		
**Income earned within the past 12 months**	less than 4'500 CHF	4.30%	3.00%	0.224	
	4500 CHF - 10'699 CHF	4,3%	3.00%		
	10'700 CHF - 15'199 CHF	8,7%	3.00%		
	15'200 CHF - 22'299 CHF	4,3%	3.00%		
	22'300 CHF - 31'249 CHF	8,7%	3.00%		
	31'250 CHF - 44'599 CHF	8,7%	3.00%		
	44'599 CHF - 66'999 CHF	13.00%	12.10%		
	67'000 CHF - 88'999 CHF	8,7%	9.10%		
	89'000 and greater	13.00%	39.40%		

	I don't know	17,4%	6.10%		
	No response	4,3%	15.20%		
		[Mean ± SD]	[Mean ± SD]		
**Subjective socioeconomic status (MacArthur)**	*N*=	20	31		
	SES Ladder	5.35±2.08	6.68±1.96	0.026	*
	N=	21	30		
	SES Comunity ladder	6.57±2.16	7.13±1.78	0.346	

* $p<0.05$; two-tailed t-test; all other t-tests non-significant at threshold of $p<0.05$
CD=conduct disorder; TD=typically developing adolescents
Education ranking according to ISCED97
Mann-Whitney *U* test used due to ordinal data

3 Medication of Adolescents With Conduct Disorder (N=35) and Typically Developing Controls (N=39) at MRI Session

		CD	TD
		N=35	*N*=39
**ADHD medication**	Methylphenidate	6	0
	Atomoxetine	1	0
**Pain medication**	Paracetamol	1	0
**Depression/bipolar disorder medication**	Quetiapine	1	0
	Valproate	1	0
**Allergy medication**	Montelukast	0	1
	Antihistamines	0	1

CD=conduct disorder; TD=typically developing adolescents
Missing data are due to time constraints

4 Button Box Responses

In the course of the study we faced technical problems with the recording of button #3 of the button box used to record the responses resulting in missing data for six controls and two patients (within the participants included in the present paper) for button number three only. Each file affected by this problem was individually evaluated with regards to whether the missing responses consistently represented correct answers. Only if we were certain that we are able to interpret the missing answers correctly, we proceeded with including the respective file into further MRI analyses (all other data were discarded).

5 In-Scanner Performance (Accuracy, Reaction Times) for Adolescents With Conduct Disorder (N=39) and Typically Developing Controls (N=39)

			CD Mean ± SD	TD Mean ± SD
**Reaction times [ms]**	Negative prime			
		Congruent	758.8 [98.8]	739.3 [101.3]
		Incongruent	857.9 [115.5]	848.2 [99.3]
	Neutral prime			
		Congruent	767.9 [96.1]	749.0 [99.7]
		Incongruent	872.5 [107.4]	852.2 [111.2]
**Accuracy [raw scores]**	Negative prime			
		Congruent	46.6 [2.8]	48.0 [2.3]
		Incongruent	43.2 [3.8]	44.8 [3.6]
	Neutral prime			
		Congruent	47.0 [2.9]	48.6 [1.5]
		Incongruent	43.9 [3.6]	45.3 [3.6]

CD=conduct disorder; TD=typically developing adolescents

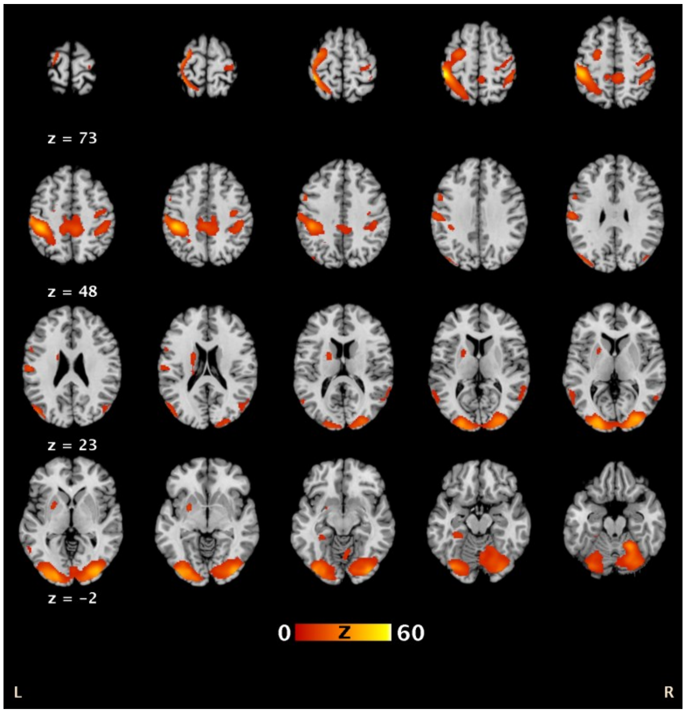

Supplementary Figure 1. Statistical parametric maps depicting the main effect of *task* during affective Stroop task processing (red-yellow) (p<0.05, FWE).
